# Estimation of Additive and Dominance Genetic Effects on Body Weight, Carcass and Ham Quality Traits in Heavy Pigs

**DOI:** 10.3390/ani11020481

**Published:** 2021-02-11

**Authors:** Valentina Bonfatti, Roberta Rostellato, Paolo Carnier

**Affiliations:** Department of Comparative Biomedicine and Food Science, University of Padova, Viale dell’Università 16, 35020 Legnaro (PD), Italy; valentina.bonfatti@unipd.it (V.B.); r.rostellato@gmail.com (R.R.)

**Keywords:** ham quality, nonadditive genetic effects, dominance variance, additive variance

## Abstract

**Simple Summary:**

The response to genetic selection in animal populations depends on both additive and nonadditive (e.g., dominance) effects. Neglecting nonadditive effects in genetic evaluations, when they are relevant, may lead to an overestimation of the genetic progress achievable. Our study evidenced that dominance effects influence the prediction of the total genetic progress achievable in heavy pigs, for growth, carcass, fresh ham and dry-cured ham seasoning traits, and indicated that neglecting nonadditive effects leads to an overestimation of the additive genetic variance. However, goodness of fit and ranking of breeding candidates obtained by models including litter and dominance effects simultaneously were not different from those obtained by models including only litter effects. Consequently, accounting for litter effects in the models for genetic evaluations, even when neglecting dominance effects, would be sufficient to prevent possible consequences arising from the overestimation of the genetic variance, with no repercussions on the ranking of animals and on accuracy of breeding values, ensuring at the same time computational efficiency.

**Abstract:**

Neglecting dominance effects in genetic evaluations may overestimate the predicted genetic response achievable by a breeding program. Additive and dominance genetic effects were estimated by pedigree-based models for growth, carcass, fresh ham and dry-cured ham seasoning traits in 13,295 crossbred heavy pigs. Variance components estimated by models including litter effects, dominance effects, or both, were compared. Across traits, dominance variance contributed up to 26% of the phenotypic variance and was, on average, 22% of the additive genetic variance. The inclusion of litter, dominance, or both these effects in models reduced the estimated heritability by 9% on average. Confounding was observed among litter, additive genetic and dominance effects. Model fitting improved for models including either the litter or dominance effects, but it did not benefit from the inclusion of both. For 15 traits, model fitting slightly improved when dominance effects were included in place of litter effects, but no effects on animal ranking and accuracy of breeding values were detected. Accounting for litter effects in the models for genetic evaluations would be sufficient to prevent the overestimation of the genetic variance while ensuring computational efficiency.

## 1. Introduction

Current genetic evaluations in pigs make use of additive genetic effects. However, the availability of estimates of nonadditive effects may increase the accuracy of prediction of breeding values [1], improve mate allocation procedures between candidates for selection [1,2], and facilitate the design of appropriate crossbreeding or purebred breeding schemes [2]. As pigs are a litter bearing species with a large expression of dominance relationships, the use of dominance models may significantly improve the accuracy of genetic evaluations, particularly when prediction of genetic merit of purebred breeding candidates is based on phenotypic information of full-sib families of crossbred individuals. The lack of informative pedigrees, such as large full-sib families, the complexity of calculations, and the difficulty in using dominant values in practice for mate allocation, make the estimation of nonadditive effects difficult [3,4]. A shortcoming of pedigree-based estimates of nonadditive effects derives from the confounding between common environmental and additive genetic effects, as dominance effects are estimated based on the combination of sire and dam and may largely coincide with litter effects [3,4]. The use of genomic information can disentangle these components because, while pedigree-based models for dominance are based on “expected” dominant relationships, genomic models are based on “observed” heterozygotes [4]. However, a major obstacle is the need of extensive data sets with genotypes and phenotypes, which are not always available [4].

Several pedigree-based studies indicated that nonadditive genetic components (e.g., dominance effects) can account for a variable proportion of the phenotypic variation in quantitative traits in a number of species [5,6,7,8]. In those studies, fitting dominance effects in statistical models resulted generally in a decrease in the estimated additive genetic variance and, consequently, in the heritability (h^2^), whereas the residual variance remained either unchanged or increased slightly. As a consequence, the predicted genetic response achievable by a breeding program may be overestimated when dominance genetic effects are neglected.

In Italy, the pig industry relies mostly on heavy pig farming where animals are fed in restricted conditions and slaughtered at 160 kg body weight (BW) and at no less than 9 months of age in order to comply with specifications of Protected Designation of Origin (PDO) dry-cured ham production [9]. Estimates of nonadditive genetic effects have been reported for a few traits and pig populations, showing that estimates are population-dependent [10]. No estimates are available for heavy pigs or ham quality traits. While the genomic reference population is still under development, an extensive dataset of phenotypic records measured on crossbred pigs within the sib-testing program of the C21 Goland sire line (Gorzagri, Fonzaso, Italy) is available.

The present study attempted to investigate the pedigree-based contribution of the additive and dominance variances to the phenotypic variation of 50 traits in crossbred heavy pigs. Traits included average daily gain, BW, carcass traits, composition of raw ham subcutaneous fat, raw ham quality traits, and ham weight losses during curing. This study provides for the first time estimates of dominance genetic effects in heavy pigs and on ham quality traits.

## 2. Materials and Methods

### 2.1. Animals

Observations used in this study were from 13,295 crossbred finishing pigs produced in the sib-testing program of the C21 Goland sire line (Gorzagri, Fonzaso, Italy). Besides growth and feed efficiency, the breeding goal of the sire line is focused on the quality of dry cured ham evaluated at the crossbred level [11,12]. Selection of C21 breeding candidates is performed using estimates of genetic merit obtained from their own phenotypes for growth performance and from phenotypic data on carcass and ham quality provided by a group of crossbred half-sibs raised in the testing farm under commercial conditions. In the testing farm, semen of C21 nucleus boars is used to inseminate a group of crossbred sows in order to produce, for each boar, families of approximately 35 crossbred piglets which are paternal half-sibs of C21 purebred breeding candidates. Crossbred sows originate from a cross involving boars of a synthetic line, derived from Large White and Pietrain breeds, and sows of a Large White line selected for maternal ability and prolificacy. In the testing farm, crossbred piglets are raised and fattened under consistent conditions and feeding strategies [13], which are comparable to those used in heavy pig farming [14]. Crossbred pigs are all slaughtered, in groups of 70 animals each, at the same abattoir (Montorsi, Correggio, Italy). Age at slaughter is constrained to a minimum of 9 months by guidelines of Parma ham production [9]. After slaughter, hams are removed from both carcass halves and dry-cured for 12 months following the Parma ham PDO specification [9]. The crossbred sib-testing program ensures the availability, for the genetic evaluation program of the sire line, of phenotypic information that are (i) specific of traits measurable only after slaughter, (ii) measured on crossbred animals owning the same genetic background of pigs originated by C21 boars in farrow-to-feeder or farrow-to-finish commercial farms, and (iii) affected by nongenetic influences that are comparable to those arising in commercial farms.

### 2.2. Carcass Traits

Final BW was adjusted to 270 d (BW270; kg) using individual linear regressions of BW on age estimated from six BW measures (at 60, 90, 135, 180, 245 d of age and the day before slaughter). Fat O-Meater (Carometec, Soeborg, Denmark) measures of carcass backfat and loin depth were used to estimate carcass lean meat content, as detailed in a previous study [13].

Measures of killing out percentage, average weight of the raw trimmed hams and weight of raw hams as a percentage of carcass weight were also available. All left thighs of crossbred pigs were further examined for raw and dry-cured ham quality traits, iodine number (IOD) and fatty acid (FA) composition of subcutaneous fat.

### 2.3. Traits Assessed on Trimmed Raw Hams

Ham subcutaneous fat depth was measured in the proximity of *semimembranosus* and *quadriceps femoris* muscles [11]. Hams were scored by a trained expert, using a linear grading system, for round shape (0: low roundness to 4: high roundness), subcutaneous fat depth (−4: low depth to 4: high depth), marbling of visible muscles of the thigh (0: low to 4: high), muscle color (−4: pale to 4: dark), and veining (visible blood vessels; 0: low to 4: high) [11]. A sample of subcutaneous fat was collected from each raw ham to assess IOD and FA composition.

### 2.4. Assessment of Iodine Number and Fatty Acid Composition of Subcutaneous Fat of Raw Hams

In agreement with official analytical procedures used by Parma ham consortium, IOD was assessed analytically on 1455 samples. Homogenized fat (30 g) was melted at 100 °C for 40 min, filtered with a paper filter and poured with anhydrous sodium sulphate to remove residual moisture. Samples were then heated at 100 °C for 30 min. An aliquot of 0.4 g was used for the determination of IOD using the Wijs method [15].

An aliquot of 5 mg of melted fat was diluted in 2 mL of N-heptane. Trans-methylation was carried out using 100 µL of Na-Methoxide and 150 µL of oxalic acid. Gas chromatography was performed on an automated apparatus (GC Shimadzu 17A, Kyoto, Japan) equipped with a flame ionization detector and a Supelco Omegowax 250 type capillary column (30 m × 0.25 mm ID; Supelco, Bellafonte, PA, USA). The operating conditions were as follows: injector temperature 260 °C, detector temperature 260 °C, helium flow 0.8 mL/min (linear velocity: 22 cm/s), thermostatic chamber program equal to 140 °C (initial isotherm) with an increase of 4 °C/min until achievement of a final isotherm of 220 °C. Fatty acids were identified by comparing their retention times to those of a mixture of FA methyl ester standards (Mix C4-24, 18919-1AMP, Supelco, Bellafonte, PA, USA). Results were expressed as the percentage of individual FA or of groups of FA in fat. Only data for the major groups of FA, individual FA representing at least 1% of fat, and ω3 FA were considered in this study.

### 2.5. Infrared Predictions of Fatty Acid Composition of Ham Subcutaneous Fat

Prediction of the percentage of C18:2n6, C18:0, ω6 FA and PUFA, of the MUFA to PUFA ratio and IOD was obtained for all samples of raw ham subcutaneous fat by near-infrared spectroscopy. Reflectance spectra were collected on a homogenized sample of the trimmed subcutaneous fat. Acquisition of the infrared spectra was performed using a Foss NIRSystem 5000 (Foss NIRSystem, Silver Spring, MD, USA) with a wavelength range of 1100–2500 nm. Prediction equations were developed through the years [16]. Such equations are very accurate, with values of R^2^ in cross-validation greater than 85% and have been used to provide phenotypes for genetic evaluations of C21 boars since 2006 [12].

### 2.6. Dry-Cured Ham Traits

Dry-cured hams were manufactured through a process that took 368 ± 4 d to complete. The three major steps (salting, resting, and curing) occurring during processing have been detailed earlier [11]. The salting phase lasted 23 d. After removing salt residues, hams were stored in resting rooms for approximately 70 d. After resting, hams were transferred to the curing phase, where they remained until the end of the dry-curing process (12 months). Left hams were weighted at the beginning and at the end of each processing stage. Measures of weight loss (%) at 23 d (end of salting), 90 d (end of resting), 12 months (end of dry-curing) and weight loss from 23 to 90 d, from 90 d to 12 months, and from 23 d to 12 months were calculated.

### 2.7. Pedigree Information

Pedigree information was available for all crossbred pigs and for all purebred C21 Goland boars, whereas only the parents and grandparents were known for the dams of the crossbred finishing pigs. Additive relationships were computed on the basis of a minimum of six generations of known ancestors. Sire and dam of crossbred pigs were unrelated.

### 2.8. Statistical Analysis

Sex and slaughter group effects were tested in preliminary analyses and were significant for all traits (*p* < 0.05), hence they were included in the models for estimation of (co)variance components. (Co)variance components were estimated using AIRemlF90 software [17] using univariate linear mixed models:

Model 0 (M-0): **y** = **Xb** + **Wa** + **Zg** + **e**, Model 1 (M-L): **y** = **Xb** + **Wa** + **Zg** + **Uc** + **e**, Model 2 (M-D): **y** = **Xb** + **Wa** + **Zg** + **Vf** + **e**, Model 3 (M-LD): **y** = **Xb** + **Wa** + **Zg** + **Uc** + **Vf** + **e**,

where **y** is a vector of observed phenotypes for one trait; **b** is a vector of nongenetic fixed effects which included sex (female and castrated male) and slaughter group effects, **a** is a random vector of additive genetic effects, **g** is a random vector of social group (animals grouped together in the same pen) effects, **c** is a random vector of litter effects, **f** is a random vector of dominance effects, **e** is a vector of random residuals, and **X**, **W**, **Z**, **U**, and **V** are incidence matrices relating **b**, **a**, **g**, **c**, and **f** to **y**, respectively. Unlike other dominance studies [13], inbreeding effects were not accounted for in models because sires and dams of crossbred pigs were unrelated.

Number of records, social groups, and families was variable across traits because phenotyping procedures did not begin simultaneously for all traits. In addition, the number of samples measured for IOD and FA composition and for dry-curing traits was considerably lower than the one for the other traits (Table A1) because analytical measures of fat quality, unlike their infrared predictions, were part of a specific research project and were not assessed routinely. The structure of the data used in this study is described in Table 1. Assumptions on the probability distributions of social group effects, litter, and residuals were:

**g**~*N* (**0**, **I**σ^2^_g_), **c**~*N* (**0**, **I**σ^2^_c_), and **e**~*N* (**0**, **I**σ^2^_e_),

where *N* ( ) indicates a normal distribution, **I** is an identity matrix of appropriate order, and σ^2^_g_, σ^2^_c_, and σ^2^_e_ are variance components for social group effects, litter, and residuals, respectively. In all models, additive genetic effects were assumed to be generated from the following probability distribution:

**a**~*N* (**0**, **A**σ^2^_a_),

where **A** is the numerator relationship matrix and σ^2^_a_ is the variance of additive genetic effects.

In models including nonadditive genetic effects, such effects were assumed to be generated from the following distribution:

**d**~*N* (**0**, **D**σ^2^_d_),

where **D** is the dominance relationship matrix. Contributions of σ^2^_a_, σ^2^_d_, σ^2^_c_, σ^2^_g_, and σ^2^_e_ to total phenotypic variance (σ^2^_P_) were also calculated. Total phenotypic variance was calculated as:

σ^2^_P_ = σ^2^_a_ + σ^2^_d_ + σ^2^_c_ + σ^2^_g_ + σ^2^_e_

To evaluate the relative importance of litter and dominance effects, the proportion of σ^2^_c_ to σ^2^_P_ (**c^2^**) was obtained for M-L and M-LD, and the proportion of σ^2^_d_ to σ^2^_P_ (**d^2^**) was obtained for M-D and M-LD. The percentage difference in c^2^ (Δc^2^%) and d^2^ (Δd^2^%) obtained by M-LD as compared to M-L and M-D, respectively, was also calculated. The magnitude of dominance effects was evaluated by the ratio of σ^2^_d_ to the total genetic variance (**D%**), calculated as σ^2^_d_/(σ^2^_a_ + σ^2^_d_).

The Akaike information criterion (AIC) was used for model pairwise comparison. When comparing models differing in the number of parameters, the parsimonious model was considered to be significantly better if its AIC was more than 2 units lower than the AIC of the complex model. Models M-L and M-D were compared by their relative likelihood. The relative likelihood of Model M-L with respect to Model M-D was calculated as exp((AIC_M−D_ − AIC_M−L_)/2) and can be interpreted as the probability that Model M-L is as good as Model M-D in minimizing the information loss.

## 3. Results and Discussion

Number of records and descriptive statistics for the investigated traits are reported in Table A1. On average, BW270 was 167 ± 15 kg, in compliance with the specification for PDO dry-cured ham production [9], which requires a minimum body weight and age at slaughter (160 kg and 270 d, respectively) to ensure optimal body tissue composition for dry-curing. Additional requirements include a minimum thickness (15 mm) and maximum IOD and linoleic acid content on total FA (70 and 15%, respectively) of ham subcutaneous fat [9].

### 3.1. Estimates of Variance Components

All models converged, except for M-D and M-LD for the ratio of the sum of SFA and MUFA to PUFA. Table 2 shows the average contribution of variance components to σ^2^_P_ obtained with the four models across traits. Estimates of σ^2^_a_, σ^2^_g_, and σ^2^_e_ obtained with M-0 represented on average 44%, 4%, and 52% of σ^2^_P_, respectively. When litter effects were included in the model (M-L, i.e., the model currently used in genetic evaluations), no substantial change in σ^2^_g_ occurred, but σ^2^_e_ slightly increased compared to the estimates obtained with M-0, whereas σ^2^_a_ decreased on average by 10%. This suggested that there is confounding between litter and additive genetic effect, as observed also previously [13]. In agreement with our results, the direct additive genetic variance for daily gain in Large White gilts, when ignoring litter effects, was of magnitude similar to the sum of litter plus additive variance when both these sources of variation were taken into account in the analysis [18]. As a consequence, ignoring contributions of litter effects to the overall variance inflated the estimated additive genetic variance, resulting in biased estimates of genetic parameters.

Group variance estimated with M-0 ranged from 0% (for the ratio of the sum of SFA and MUFA to PUFA) to 8% of σ^2^_P_ and it remained constant across models, suggesting that there was no confounding between group and other effects. Pigs were assigned to pens randomly, as to minimize the probability of forming groups constituted by individuals from the same litter. This enabled separation of group and litter variance in the estimation process. Residual variance estimated by M-0 represented on average 52% of σ^2^_P_ and its proportion to σ^2^_P_ ranged from 19% to 73%. Across traits, it slightly increased (by 2%) in M-L, and decreased (by 9%) in M-D and M-LD, compared to M-0. For five traits (SFA, Unsaturated FA/SFA, C14:0, C16:0 and ω3), σ^2^_c_ and σ^2^_d_ were not different from 0.

### 3.2. Heritability Estimates

Estimates of σ^2^_a_ and h^2^ obtained with M-0 are reported in Table 3. The SE of σ^2^_a_ ranged from 2% to 7% of the point estimate in the carcass traits, ham evaluation traits, and infrared-predicted fat composition. It was 7–13% of σ^2^_a_ for dry-curing traits and 7–17% of σ^2^_a_ in fat composition traits, with the only exception of C18:0 and C16:1, for which the SE was more than 30% of σ^2^_a_. Standard errors obtained by M-L, M-D and M-LD were of the same magnitude of those obtained by M-0 (results not reported in tables). Heritability ranged from 0.24 (for ham weight loss from resting to the end of curing, %) to more than 0.70 (for C18:0 and C16:1 contents). The SE of h^2^ estimates averaged 0.05 and ranged from 0.02 to 0.14. Values of h^2^ for BW270, backfat depth, carcass lean meat content, IOD, linoleic acid, ham subcutaneous fat depth measured in the proximity of *semimembranosus* and *quadriceps femoris* muscles, round shape, subcutaneous fat, and marbling scores were in agreement with findings of a previous study carried out using M-0 on the same traits and genetic line [13].

Weight losses during the different phases of seasoning exhibited h^2^ values ranging from 24% to 32% for percentage losses, and from 40% to 55% for losses expressed as kg. Heritability for quality traits collected during dry-curing of hams have been scarcely investigated, except for the % weight loss at first salting (7 d). Its estimates of h^2^ ranged from 0.30 to 0.61 [19,20,21] and the trait is currently used in selection plans toward the improvement of meat quality for seasoning aptitude in Italian purebred pigs. In the current study, h^2^ estimated for the % weight loss at the end of salting (23 d) was 0.29, close to the lowest estimate reported in the literature for weight loss at first salting. Pigs enrolled in this study were raised in the same farm under standardized conditions, and slaughtered at the same abattoir following standardized practices. These factors likely contributed to the generally medium-to-high h^2^ estimates.

As a consequence of the possible covariance that nonadditive genetic effects create among family members, ignoring contributions of family effects to the overall variance might inflate the estimated additive genetic variances and might result in biased estimates of genetic parameters [13]. For this reason, values of h^2^ estimated with the four models were compared. The percentage difference in h^2^ obtained by M-L, M-D, and M-LD compared to those estimated by M-0 are reported in Table 3. Across traits, h^2^ estimated with M-L was on average 9%, and up to 26%, lower than that obtained with M-0. The decrease was lower than 5% for 17 traits, but it was greater than 25% for the ratio of the sum of SFA and MUFA to PUFA, and ham % weight loss from resting to the end of curing. The lower h^2^ estimates obtained by M-L compared to M-0 were due to a decrease in additive genetic variance (Table 2). This was consistent across all the traits for which σ^2^_c_ was >0.

These results are in agreement with those obtained in another study [13] performed on the same crossbred pig population reporting that, when litter effects were neglected, h^2^ were larger than those obtained with models accounting for litter effects, as a consequence of inflated estimates of additive genetic variance. A further slight decrease in h^2^ (by 1.5 percentage points on average) was observed comparing M-L with M-D. This indicates that the inclusion of litter, dominance, or both in models for genetic evaluations is expected to have a considerable effect on h^2^ estimates, and, consequently, on the estimated genetic progress.

Results from model M-LD were very similar to those of M-L and M-D, with the exception of IOD and some of the dry-curing traits, for which h^2^ dropped further in model M-LD when compared to M-D. In agreement with our results, pedigree-based studies reported in the literature have consistently shown that fitting nonadditive effects, particularly dominance genetic effects, resulted in a remarkable decrease in the h^2^ estimates, while the residual variance either remained the same or increased slightly. Across traits, the decrease in h^2^ ranged from 3% to 53%. Similar tendencies were observed for pig longevity traits [22], for the number of kits born alive and dead, respectively [7], and for daily gain in pigs [8].

### 3.3. Confounding between Litter and Dominance Variance

Estimates of the proportion of σ^2^_c_ to σ^2^_P_ (c^2^), and of σ^2^_d_ to σ^2^_P_ (d^2^) obtained by M-L and M-D, respectively, are reported in Table 4. Across traits, c^2^ was on average 0.025 (see also Table 2) and ranged from 0 to 0.07. On average, d^2^ was 0.11 and ranged from 0 to 0.26. Standard errors were generally around 30% of c^2^ and d^2^ estimates for carcass traits, ham evaluation traits, and infrared predicted fat composition, whereas higher SE were obtained for FA and ham weight losses, as these traits were available for a limited number of animals (≈1450 and ≈1700 for FA and weight losses, respectively). For some of the FA and most measures of ham weight loss, the SE was as big as the estimate or greater.

The estimated σ^2^_d_ may partly contain a full-sib common environmental variance [4] and this effect should be fitted along the dominance effect. For the traits investigated in this study, for which phenotypes are measured far from the time when the litter mates share a common environment, the common environmental variance is expected to be low and the fitting of litter effects in models may be a simple way to account for nonadditive genetic effects shared by full-sib family members. Estimates of c^2^ and d^2^ varied when litter and dominance effects were fitted simultaneously in the model (M-LD). The percentage differences in c^2^ (Δc^2^%) and d^2^ (Δd^2^%) obtained by M-LD as compared to M-L and M-D, respectively, are reported in Table 4. Across all traits in which σ^2^_c_ was significantly different from zero, the proportion of σ^2^_c_ to σ^2^_P_ in M-LD was on average only 30% (ranging from 0% to 100%) of the one estimated by M-L. This indicates that a large part of σ^2^_c_ was removed when accounting for dominance effects due to confounding between litter and dominance effects.

Analogously, across the traits with reliable and non-null estimates of dominance effects, the proportion of σ^2^_d_ to σ^2^_P_ in M-LD was approximately 70% (ranging from 0% to 100%) of the one estimated by M-D. This is a further indication of the confounding between litter and dominance effects. As a consequence, a model including litter effects will account for dominance effects as well, and vice versa.

Other pedigree-based studies found similar results: common litter variance components were twice as high using models that did not contain dominance effects compared to a model containing dominance and litter effects in pigs [22]; likewise, full-sib effect of laying hens removed almost all the dominance variance when the dominance effect was not included in the model, while dominance effects explained almost all the full-sib variance when full-sib effect was not included in the statistical model [23].

### 3.4. Magnitude of Dominance Variance

Being litter and dominance effects confounded, the estimates of σ^2^_d_ obtained with M-LD might be affected by the presence in the model of the litter effect, and vice-versa. Therefore, the magnitude of dominance variance was evaluated considering estimates of σ^2^_d_ obtained from M-D. On the other hand, these estimates may be inflated as they represent both litter and dominance components. Proportion of σ^2^_d_ to total genetic variance (D%) and of σ^2^_d_ to σ^2^_a_ (Da%) obtained with M-D are reported in Table 4. Across carcass and raw ham evaluation traits, D% was on average 27.6% and ranged from 10% (for veining score) to 41% (for body weight at 270 d).

For FA, D% ranged from 0% to 44%. Dominance variance was null or negligible for SFA and individual saturated FA, but it represented approximately 40% of σ^2^_a_ for the content of PUFA, C18:1n9ct, C18:2n6, and ω6. The proportion of σ^2^_d_ to total genetic variance (D%) was on average 21% across infrared-predicted traits, ranging from 9% to 28%. Values of D% were 40% and 45% for the initial and final ham weight and ranged from 0% to 60% in ham curing weight loss traits. However, estimates for ham weight loss traits exhibited very high SE, with the exception of the weight loss from resting to the end of curing, for which D% was above 40%.

Across traits, σ^2^_d_ contributed up to 154% of σ^2^_a_ (for percentage ham weight loss from resting to the end of curing). In particular, it accounted for at least 26% of σ^2^_a_ for all carcass traits. For traits measured on raw hams, Da% ranged from 11% to 38% for the raw ham quality traits evaluated with the linear scoring system, whereas it was 19% and 35% for the measures of subcutaneous fat depth. Values of Da% reached 78% in FA composition (for ω6) and 38% for infrared predicted fat composition (for the ratio between MUFA and PUFA).

In pigs, most of the estimates of nonadditive genetic effects have been obtained for maternal traits, daily gain, and backfat thickness [24], and the large majority of the studies were performed on purebred pigs, in which dominance effects are expected to be small, as compared to crossbreds [25]. Estimates varied across studies, supporting the hypothesis that dominance effects are trait- and population-specific. Detection of dominance variance needs the locus to be segregating at intermediate gene frequency, hence population-specific dominance effects can result from differences in allele frequencies in each population [25].

In purebreds, the ratio of σ^2^_d_ to the total phenotypic variance ranged from 0.04 to 0.11 for growth traits, and between 0.02 and 0.05 for backfat thickness [24,25,26,27], hence dominance effects contributed only slightly to the phenotypic expression of the traits investigated, and their contributions were lower than the contributions of additive genetic effects. These estimates, as expected, are lower than those obtained in our study.

For growth traits, the high absolute value of σ^2^_d_, as well as the large σ^2^_d_ compared with σ^2^_a_ found in our study, agreed with previous results for growth traits in crossbred pigs [10]. Estimates of σ^2^_d_ of body weight at different ages were reported to contribute to 27–54% of σ^2^_a_, while the ratio of σ^2^_d_ to σ^2^_a_ was 1.17 for slaughter weight, 0.57 for carcass weight, 0.94 for loin eye area and it ranged from 0.57 to 1.56 for different measures of backfat thickness [10].

Despite the uncertainty of the estimates due to the limited amount of records, a recent study [28] reported ratios of dominance deviation variance to the total phenotypic variance in 22 traits related to growth rate, feed efficiency, carcass composition, meat quality, behavior, boar taint, and puberty. For many traits, the dominance deviation variance was higher in crossbreds than in purebreds, but a clear common pattern of dominance expression between groups of analyzed traits and between populations was not encountered. In that study, the ratio of dominance deviation variance to phenotypic variance in crossbreds was 0.08 for average daily gain, 0.12 for backfat thickness, 0.09 for lean meat content, and 0.14 for ham cut (kg/kg). These values are slightly lower than our estimates. To our knowledge, dominance effects have never been estimated for fat composition and dry-cured ham quality traits, but results of the current study, although associated to relatively high SE, seem to indicate that also these traits may be affected by nonadditive effects.

### 3.5. Usefulness of Including Dominance in Models for Genetic Evaluations

To determine whether including litter or dominance effects in models for genetic evaluations improved model fitting, AIC of models M-L and M-D were compared to those yielded by M-0 (Table 5). For all carcass and raw ham evaluation traits except for veining score, as well as for all the infrared predicted traits and initial and final ham weight, both M-L and M-D had a significantly better fitting than M-0. Marginal improvements were also obtained with M-L for (SFA+MUFA)/PUFA and with M-D for ham weight loss (expressed as % and kg) from resting to the end of curing. However, model fitting of M-LD was not significantly different from that of M-L and of M-D, indicating that fitting either the litter effect or the dominance effect is sufficient to account for the nonadditive components. To evaluate whether dominance effects should be included in models for genetic evaluations in place of litter effects, the AIC of model M-D was also compared to the one obtained by M-L (Table 5). For 24 traits for which the AIC of M-D was significantly lower than that of M-0, the AIC of model M-D was also slightly lower than the one of M-L. These models were also compared using their relative likelihood. In 15 out of those 24 traits, M-D had a better fitting (relative likelihood < 0.8) than M-L. These traits include carcass, raw ham evaluation traits, infrared predicted FA, and ham weight loss (expressed as % and kg) from resting to the end of curing.

Despite the small difference in AIC of M-D models compared to M-L, the Spearman’s rank correlation between the breeding values (EBV) estimated by the different models for C21 breeding candidates and for C21 nucleus boars were >0.999 in all traits (data not reported in tables). These results indicate that, although dominance variance represents a significant proportion of the total genetic variance, the ranking of breeding candidates provided by models neglecting dominance effects is very similar to the one obtainable by models in which such effects are accounted for. Very high correlations (>0.999) between EBV predicted by pedigree-based models including or not dominance effects were reported also for stature in cattle [29], harvest body weight in Coho salmon [1], and number of kits born alive, number of kits born dead, and total number of kits in rabbit [7]. In addition, the accuracy of the EBV did not improve when models accounted for dominance effects, as reported in the majority of studies [4].

Substantial dominance variation was found to affect carcass and ham quality traits. Litter and dominance effects affect the estimates of h^2^ and, if their contribution to the total genetic variance is ignored, the heritable variation and the response to selection may be incorrectly estimated. Nonadditive genetic components such as dominance effects are usually not accounted for in pedigree-based models because they tend to be confounded with the common maternal environment and they are thought to have little practical application in selection [3,4]. In addition, their estimation is computationally demanding. Currently, genetic evaluation of breeding candidates of the C21 sire line is performed for all the investigated traits with models neglecting nonadditive genetic effects, but including litter effects. Our results indicate that, for some traits, the common litter effect removes part or all of the nonadditive genetic effects when the two effects are accounted for in the model jointly. Accurate prediction of nonadditive effects may be important in selection of mates based on their specific combining abilities [3], where these nonadditive genetic effects may be exploited directly through specific mate allocation. However, specific mate allocation is not performed in commercial farms rearing crossbred finishing pigs. In such case, accounting for litter effects, even though neglecting dominance effects, in the models for genetic evaluations would be sufficient to prevent the effects arising from the overestimation of the genetic variance in a computationally efficient way. 

Nonadditive effects result from the interaction between alleles at a locus (dominance), and among alleles at different loci (epistasis). For the past 30 years, the goal of molecular quantitative genetics has been to define the genetic architecture of quantitative traits, to identify whether allelic effects are additive within and across loci, one allele is dominant over another, or the effect of a quantitative trait locus (QTL) is dependent on the genotype at another locus [30]. In quantitative genetics, partitioning genetic variance for a trait into statistical components due to additivity, dominance, and epistasis is useful for prediction and selection, even if it does not reflect the biological (or functional) effect of the underlying genes [30]. In pedigree-based estimates, while epistasis refers to the interaction among additive and dominance genetic effects (e.g., additive by additive, additive by dominance, additive by additive by dominance), dominance relationship between two given animals represents the probability that they share common pairs of alleles [31]. If two animals have the same set of parents or grandparents, it is possible that they share common pairs of alleles [31]. As a consequence, in studies performed on full-sib families, the dominance relationship matrix tends to be very similar to the incidence matrix of the common litter effect and genetic factors can be confounded with nongenetic factors such as shared environmental effects. Methods exploiting genomic information, as compared to traditional pedigree-based quantitative genetics methods, provide more accurate estimates of dominance effects [3] because the computation of the genomic dominance relationship matrix only requires knowledge about whether marker genotypes are heterozygous or not, and the estimate does not rely on probabilities of identical genotypes. As a consequence, dominance effects can be successfully disentangled from common environment effects [32]. However, accurate estimates of dominance variance need large genomic datasets (>2000 records) [25], as well as a large number of genotyped individuals per litter, in order to enable the detection of identical genotypes among individuals and dominance relationships [27]. For mating programs, genomic data can also be used to calculate genotype probabilities of hypothetical progeny resulting from possible matings between candidates [1]. These probabilities together with the estimated additive and dominance effects of marker genotypes can be used to define a set of mates that maximize performance in the future generation, if genotypes of males and females are available in the population. Compared to random mating, mate allocation can generate a further increase in the genetic response ranging between 6% and 22% [1].

## 4. Conclusions

Substantial dominance variation was found to affect carcass and ham quality traits, however, litter and dominance effects could not be disentangled. For some traits, the common litter effects removed part or all of the variance due to nonadditive genetic effects when both such effects were accounted for by the statistical model. Neglecting litter and dominance effects affected the estimates of h^2^ and, when their contribution to the phenotypic variance is ignored by models, the heritable variation and the expected response to selection may be incorrectly estimated. Accurate prediction of nonadditive effects may be important in selection of mates based on their specific combining abilities. However, specific mate allocation is not performed in commercial farms rearing crossbred finishing pigs. In such case, accounting for litter effects in place of dominance effect in the models for breeding values prediction would be sufficient to prevent possible effects arising from the overestimation of the genetic variance, with no effect on the ranking of animals and accuracy of EBV, and to ensure computational efficiency. The availability of genomic information enables the dissection of the total genetic variance into additive and nonadditive components. In the near future, the dominance contribution to the total variance in the traits investigated in this study might be re-evaluated making use of genomic information.

## Figures and Tables

**Table 1:** Structure of the data used in the study.

Item	N
Crossbred pigs	13,395
Gilts	6454
Barrows	6941
Nucleus boars of the C21 line used as sires of crossbred pigs	399
Average number of crossbreds per C21 nucleus boar	33.6
Sows used as dams of crossbred pigs	793
Average number of crossbreds per sow	16.9
Average number of crossbred litters per sow	3.2
Number of crossbred full-sib families	2495
Average size of crossbred full-sib families	5.4
Number of crossbred social groups	2061
Average size of crossbred social groups	6.5
Number of slaughter batches	200
Average size of slaughter batches	67

**Table 2:** Average contribution (%) of variance components to the total phenotypic variance estimated for the investigated traits with four models (M-0, M-L, M-D, M-LD) ^1^.

Variance Component	Model			
	M-0	M-L	M-D	M-LD
Additive	44.11	40.30	39.05	38.34
Dominance	-	-	11.28	9.75
Litter	-	2.45	-	0.78
Group	3.57	3.56	3.59	3.58
Residual	52.32	53.68	46.07	47.55

^1^ Model M-0 included the fixed effects of sex and slaughter group, and the random effects of social group and animal. In addition to the effects included in M-0, Models M-L and M-D included the random effect of the litter and the dominance effect, respectively. Model M-LD included all the effects of M-0, the random effects of litter and the dominance effect.

**Table 3:** Estimates of additive genetic variance (σ^2^_a_) and h^2^ (± SE) with Model M-0 and variation (%) in h^2^ estimates obtained by models M-L, M-D, and M-LD ^1^.

Trait	σ^2^_a_ M-0	h^2^ M-0	h^2^ M-L vs. M-0	h^2^ M-D vs. M-0	h^2^ M-LD vs. M-0
Average daily gain, kg/d	2.17 × 10^−3^ ± 1.50 × 10^−4^	0.481 ± 0.026	−16.6	−19.2	−17.6
Body weight at 270 d, kg	93.501 ± 6.488	0.457 ± 0.025	−19.9	−23.9	−22.6
Killing out percentage, %	0.483 ± 0.038	0.316 ± 0.021	−6.9	−9.5	−8.8
Backfat depth, mm	9.961 ± 0.724	0.451 ± 0.026	−16.2	−20.1	−19.0
Carcass lean meat content, %	2.069 ± 0.155	0.441 ± 0.027	−16.7	−21.1	−20.7
Loin depth, mm	10.698 ± 0.903	0.306 ± 0.023	−16.8	−21.2	−17.5
Average weight of the thighs, kg	0.548 ± 0.038	0.478 ± 0.026	−16.6	−20.4	−19.6
Raw ham percentage, %	0.421 ± 0.027	0.573 ± 0.027	−9.1	−10.6	−9.1
Raw ham traits					
Round shape score	0.255 ± 0.019	0.362 ± 0.023	−7.2	−9.6	−9.4
Marbling score	0.271 ± 0.019	0.408 ± 0.024	−7.6	−10.7	−10.4
Subcutaneous fat score	1.188 ± 0.084	0.424 ± 0.025	−10.6	−13.1	−10.6
Color score	0.699 ± 0.053	0.337 ± 0.022	−9.3	−12.6	−11.9
Veining score	0.190 ± 0.016	0.248 ± 0.019	−2.2	−3.6	−4.0
Subcutaneous fat depth, mm					
nearby *semimembranosus muscle*	10.753 ± 0.794	0.392 ± 0.024	−5.6	−8.4	−8.3
nearby *quadriceps femoris* muscle	3.21 × 10^−3^ ± 2.67 × 10^−4^	0.397 ± 0.027	−8.6	−12.0	−11.9
Fatty acid composition					
SFA, %	1.448 ± 0.220	0.589 ± 0.069	0.0	0.0	−6.4
MUFA, %	1.213 ± 0.192	0.541 ± 0.068	−9.5	−12.4	−12.4
PUFA, %	0.788 ± 0.149	0.399 ± 0.065	−18.9	−23.7	−23.7
Unsaturated FA/SFA	9.14 × 10^−3^ ± 1.35 × 10^−3^	0.603 ± 0.068	1.0	−0.8	−25.4
(SFA + MUFA)/PUFA	0.171 ± 0.035	0.408 ± 0.143	−25.0	−28.1	−27.8
C14:0, %	5.54 × 10^−3^ ± 9.56 × 10^−4^	0.476 ± 0.068	1.8	0.0	0.1
C16:0, %	0.328 ± 0.063	0.380 ± 0.064	0.0	0.0	0.0
C18:0, %	0.679 ± 0.091	0.788 ± 0.070	−0.5	−0.4	−0.4
C16:1, %	0.068 ± 0.009	0.793 ± 0.071	0.2	−6.3	−5.3
C18:1n9ct, %	0.889 ± 0.151	0.485 ± 0.068	−19.7	−24.1	−24.1
C18:1n11trans, %	0.039 ± 0.006	0.562 ± 0.068	−2.6	−2.2	−2.2
C18:2n6, %	0.634 ± 0.119	0.410 ± 0.065	−17.7	−22.4	−22.4
ω3, %	2.72 × 10^−3^ ± 6.38 × 10^−4^	0.250 ± 0.054	0.9	0.0	−0.9
ω6, %	0.658 ± 0.125	0.399 ± 0.065	−21.8	−27.0	−27.0
Iodine number	3.156 ± 0.518	0.525 ± 0.069	−1.5	−2.4	−17.9
Infrared predictions					
Iodine number	2.315 ± 0.164	0.453 ± 0.026	−8.3	−10.4	−9.6
C18:2n6, %	0.723 ± 0.056	0.471 ± 0.029	−9.4	−12.3	−12.0
C18:0, %	0.451 ± 0.031	0.616 ± 0.031	−3.9	−4.2	−4.0
ω6, %	0.755 ± 0.058	0.467 ± 0.029	−9.3	−12.3	−12.3
PUFA, %	0.909 ± 0.070	0.467 ± 0.029	−9.3	−12.2	−12.1
MUFA/PUFA, %	0.054 ± 0.004	0.475 ± 0.028	−11.0	−14.6	−14.3
Dry-curing traits					
Initial ham weight, kg	0.516 ± 0.087	0.499 ± 0.067	−21.4	−24.3	−23.2
Final ham weight, kg	0.296 ± 0.055	0.396 ± 0.062	−23.9	−26.5	−25.9
Ham weight loss, %					
At the end of salting	0.088 ± 0.019	0.290 ± 0.056	0.2	−0.6	−13.8
From salting to the end of resting	0.229 ± 0.054	0.248 ± 0.054	−15.4	−17.2	−15.4
From resting to the end of curing	0.650 ± 0.153	0.243 ± 0.053	−26.2	−39.2	−44.6
At the end of resting	0.488 ± 0.101	0.318 ± 0.058	−12.2	−15.2	−14.9
At the end of dry-curing	1.433 ± 0.299	0.307 ± 0.057	−5.1	−8.4	−16.0
From salting to end of dry-curing	1.085 ± 0.237	0.280 ± 0.055	−6.4	−9.9	−15.9
Ham weight loss, kg					
At the end of salting	2.28 × 10^−3^ ± 4.26 × 10^−4^	0.401 ± 0.063	0.0	−1.0	−1.1
From salting to the end of resting	8.73 × 10^−3^ ± 1.55 × 10^−3^	0.448 ± 0.066	1.5	1.2	0.0
From resting to the end of curing	0.016 ± 0.003	0.396 ± 0.063	−18.6	−26.6	−31.7
At the end of resting	0.019 ± 0.003	0.537 ± 0.068	0.6	−1.3	−2.4
At the end of dry-curing	0.065 ± 0.011	0.550 ± 0.069	−1.9	−5.8	−6.8
From salting to end of dry-curing	0.045 ± 0.008	0.516 ± 0.068	−0.3	−4.4	−13.9

^1^ Model M-0 included the fixed effects of sex and slaughter group, and the random effects of the social group and animal additive genetic effects; Model M-L: as M0 + litter effects; M-D: as M0 + dominance effects; Model M-LD: as M0 + litter and dominance effects.

**Table 4:** Ratios (± SE) of litter (c^2^) and dominance (d^2^) to total variance obtained by M-L and M-D, of dominance to total genetic variance (D%) and to additive genetic variance (Da%) obtained by M-D, and percentage difference in c^2^ (Δc^2^%) and d^2^ (Δd^2^%) estimated by M-LD as compared to M-L and M-D, respectively ^1^.

Trait	c^2^ M-L	d^2^ M-D	Δc^2^%	Δd^2^%	D% M-D	Da% M-D
Average daily gain, kg/d	0.044 ± 0.008	0.182 ± 0.034	−25.1	−73.5	31.9	46.9
Body weight at 270 d, kg	0.057 ± 0.008	0.245 ± 0.035	−56.9	−41.7	41.3	70.3
Killing out percentage, %	0.016 ± 0.007	0.074 ± 0.031	−98.5	−8.1	20.6	25.9
Backfat depth, mm	0.043 ± 0.008	0.187 ± 0.036	−63.9	−34.6	34.2	51.9
Carcass lean meat content, %	0.042 ± 0.009	0.187 ± 0.038	−87.9	−11.3	35.0	53.8
Loin depth, mm	0.041 ± 0.009	0.170 ± 0.037	−12.6	−86.7	41.4	70.6
Average weight of the thighs, kg	0.052 ± 0.008	0.223 ± 0.035	−70.8	−28.0	37.0	58.6
Raw ham percentage, %	0.036 ± 0.007	0.149 ± 0.031	−0.1	−99.9	22.5	29.0
Raw ham traits						
Round shape score	0.021 ± 0.007	0.093 ± 0.030	−86.6	−12.7	22.2	28.6
Marbling score	0.022 ± 0.007	0.103 ± 0.031	−74.5	−20.6	22.0	28.2
Subcutaneous fat score	0.032 ± 0.007	0.133 ± 0.031	0.0	−100.0	26.5	36.0
Color score	0.026 ± 0.007	0.112 ± 0.031	−73.2	−25.6	27.6	38.2
Veining score	0.005 ± 0.007	0.027 ± 0.029	−80.1	−0.4	10.2	11.3
Subcutaneous fat depth, mm						
nearby *semimembranosus* muscle	0.014 ± 0.007	0.070 ± 0.030	−99.7	−1.6	16.3	19.4
nearby *quadriceps femoris* muscle	0.028 ± 0.009	0.124 ± 0.038	−96.1	−3.6	26.2	35.4
Fatty acid composition						
SFA, %	0.000 ± 0.000	0.000 ± 0.000	ne	ne	0.0	0.0
MUFA, %	0.027 ± 0.029	0.126 ± 0.120	−100.0	0.0	21.0	26.6
PUFA, %	0.044 ± 0.033	0.198 ± 0.135	−100.0	0.0	39.4	65.0
Unsaturated FA/SFA	0.000 ± 0.000	0.009 ± 0.003	ne	−27.7	1.5	1.5
(SFA + MUFA)/PUFA	0.073 ± 0.056	nc	ne	ne		nc
C14:0, %	0.000 ± 0.000	0.000 ± 0.000	ne	ne	0.0	0.0
C16:0, %	0.000 ± 0.000	0.000 ± 0.000	ne	ne	0.0	0.0
C18:0, %	0.002 ± 0.023	0.006 ± 0.099	−99.9	−0.5	0.7	0.7
C16:1, %	0.000 ± 0.000	0.094 ± 0.114	ne	−60.4	11.2	12.6
C18:1n9ct, %	0.051 ± 0.032	0.229 ± 0.133	−100.0	0.0	38.4	62.4
C18:1n11trans, %	0.008 ± 0.025	0.024 ± 0.103	−99.9	−0.3	4.2	4.4
C18:2n6, %	0.041 ± 0.033	0.190 ± 0.136	−100.0	0.0	37.3	59.6
ω3, %	0.000 ± 0.000	0.000 ± 0.001	ne	ne	0.1	0.1
ω6, %	0.050 ± 0.034	0.227 ± 0.139	−100.0	0.0	43.8	78.0
Iodine number	0.005 ± 0.028	0.028 ± 0.119	−100.0	129.5	5.1	5.4
Infrared predictions						
Iodine number	0.025 ± 0.007	0.107 ± 0.032	−53.0	−45.6	20.9	26.4
C18:2n6, %	0.028 ± 0.008	0.123 ± 0.036	−86.8	−12.6	22.9	29.7
C18:0, %	0.016 ± 0.007	0.057 ± 0.030	−3.4	−95.4	8.9	9.7
ω6, %	0.027 ± 0.008	0.122 ± 0.036	−99.4	−0.1	22.9	29.7
PUFA, %	0.027 ± 0.008	0.121 ± 0.035	−94.3	−5.4	22.7	29.4
MUFA/PUFA, %	0.034 ± 0.008	0.154 ± 0.036	−79.4	−17.4	27.6	38.1
Dry-curing traits						
Initial ham weight, kg	0.061 ± 0.027	0.255 ± 0.115	−46.8	−51.5	40.3	67.5
Final ham weight, kg	0.060 ± 0.027	0.240 ± 0.116	−36.3	−59.9	45.2	82.4
Ham weight loss, %						
At the end of salting	0.000 ± 0.000	0.004 ± 0.094	ne	135.3	1.3	1.3
From salting to the end of resting	0.027 ± 0.025	0.104 ± 0.104	−0.1	−100.0	33.7	50.8
From resting to the end of curing	0.047 ± 0.026	0.228 ± 0.114	−100.0	10.2	60.6	154.1
At the end of resting	0.026 ± 0.024	0.113 ± 0.103	−80.1	−18.1	29.5	41.8
At the end of dry-curing	0.011 ± 0.023	0.060 ± 0.099	−100.0	88.9	17.5	21.3
From salting to end of dry-curing	0.013 ± 0.023	0.065 ± 0.101	−100.0	58.9	20.5	25.7
Ham weight loss, kg						
At the end of salting	0.000 ± 0.020	0.009 ± 0.089	91.6	−0.5	2.2	2.2
From salting to the end of resting	0.000 ± 0.000	0.000 ± 0.000	ne	ne	0.0	0.0
From resting to the end of curing	0.042 ± 0.025	0.209 ± 0.112	−100.0	17.0	41.8	72.0
At the end of resting	0.000 ± 0.000	0.014 ± 0.087	ne	0.2	2.7	2.7
At the end of dry-curing	0.006 ± 0.020	0.060 ± 0.093	−51.5	−0.6	10.4	11.7
From salting to end of dry-curing	0.000 ± 0.000	0.043 ± 0.093	ne	115.1	7.9	8.6

^1^ Model M-0 included the fixed effects of sex and slaughter group, and the random effects of the social group and animal additive genetic effects; Model M-L: as M0 + litter effects; M-D: as M0 + dominance effects; Model M-LD: as M0 + litter and dominance effects; nc: not converged; ne: not estimable.

**Table 5:** Differences in Akaike information criterion (AIC) of models and relative likelihood (RL) of Model M-L with respect to Model M-D (for M-D models that were significantly different from M-0 and with AIC lower than that of the M-L model). Models are assumed to be significantly different (*) for differences in AIC < −2 ^1,2^.

Trait	M-L vs. M-0	M-D vs. M-0	M-LD vs. M-L	M-LD vs. M-D	M-D vs. M-L	RL
Average daily gain, kg/d	−35.67 *	−34.31 *	1.84	0.48	1.36	
Body weight at 270 d, kg	−65.09 *	−65.49 *	0.86	1.26	−0.40	0.82
Killing out percentage, %	−3.13 *	−4.41 *	0.76	2.04	−1.28	0.53
Backfat depth, mm	−32.13 *	−32.70 *	1.13	1.70	−0.57	0.75
Carcass lean meat content, %	−28.69 *	−30.29 *	0.36	1.97	−1.60	0.45
Loin depth, mm	−24.99 *	−23.80 *	1.97	0.78	1.19	
Average weight of the thighs, kg	−51.35 *	−52.50 *	0.57	1.72	−1.15	0.56
Raw ham percentage, %	−30.51 *	−28.55 *	2.00	0.04	1.96	
Raw ham traits						
Round shape score	−8.28 *	−8.75 *	1.51	1.99	−0.47	0.79
Marbling score	−9.60 *	−11.08 *	0.74	2.22	−1.48	0.48
Subcutaneous fat score	−21.12 *	−19.61 *	2.00	0.49	1.51	
Color score	−12.49 *	−12.91 *	1.52	1.94	−0.42	0.81
Veining score	1.35	1.06	1.77	2.07	−0.30	0.86
Subcutaneous fat depth, mm						
nearby *semimembranosus* muscle	−2.15 *	−3.54 *	0.62	2.00	−1.38	0.50
nearby *quadriceps femoris* muscle	−10.47 *	−11.25 *	1.21	2.00	−0.78	0.68
Fatty acid composition						
SFA, %	2.00	2.00	4.37	4.37	0.00	
MUFA, %	0.98	0.70	1.73	2.00	−0.27	0.87
PUFA, %	0.02	−0.33	1.65	2.00	−0.35	0.84
Unsaturated FA/SFA	2.01	2.23	2.58	2.36	0.22	
(SFA + MUFA)/PUFA	−2.04 *	nc	nc	nc	nc	nc
C14:0, %	2.02	2.00	2.10	2.12	−0.02	0.99
C16:0, %	2.00	2.00	2.00	2.00	0.00	
C18:0, %	1.99	2.00	2.01	2.00	0.01	
C16:1, %	3.03	1.19	0.25	2.08	−1.83	0.40
C18:1n9ct, %	−1.17	−1.60	1.57	2.00	−0.43	0.81
C18:1n11trans, %	1.90	1.94	2.04	2.00	0.04	
C18:2n6, %	0.28	−0.09	1.63	2.00	−0.37	0.83
ω3, %	2.00	2.00	2.02	2.02	0.00	
ω6, %	−0.52	−0.98	1.54	2.00	−0.46	0.80
Iodine number	1.97	1.94	10.22	10.24	−0.03	0.99
Infrared predictions						
Iodine number	−11.38 *	−11.42 *	1.79	1.83	−0.05	0.98
C18:2n6, %	−11.45 *	−12.04 *	1.39	1.99	−0.59	0.74
C18:0, %	−3.93 *	−2.24 *	2.05	0.37	1.69	
ω6, %	−10.88 *	−11.70 *	1.18	2.00	−0.82	0.66
PUFA, %	−10.79 *	−11.45 *	1.34	2.00	−0.66	0.72
MUFA/PUFA, %	−19.26 *	−20.78 *	0.57	2.09	−1.52	0.47
Dry-curing traits						
Initial ham weight, kg	−4.62 *	−4.59 *	1.92	1.89	0.03	
Final ham weight, kg	−4.09 *	−3.51 *	2.13	1.54	0.58	
Ham weight loss, %						
At the end of salting	2.00	2.00	2.72	2.73	0.00	
From salting to the end of resting	0.74	0.93	2.00	1.81	0.19	
From resting to the end of curing	−1.95	−2.76 *	1.24	2.04	−0.81	0.67
At the end of resting	0.80	0.72	1.92	2.00	−0.08	0.96
At the end of dry-curing	1.77	1.64	2.12	2.24	−0.13	0.94
From salting to end of dry-curing	1.69	1.59	2.02	2.12	−0.11	0.95
Ham weight loss, kg						
At the end of salting	2.00	1.99	1.99	2.00	−0.01	1.00
From salting to the end of resting	2.01	2.01	1.99	1.99	0.00	
From resting to the end of curing	−1.34	−2.37 *	1.07	2.10	−1.03	0.60
At the end of resting	2.00	1.97	2.12	2.15	−0.03	0.99
At the end of dry-curing	1.93	1.56	1.74	2.11	−0.37	0.83
From salting to end of dry-curing	2.00	1.79	2.58	2.79	−0.21	0.90

^1^ Model M-0 included the fixed effects of sex and slaughter group, and the random effects of the social group and animal additive genetic effects; Model M-L: as M0 + litter effects; M-D: as M0 + dominance effects; Model M-LD: as M0 + litter and dominance effects. ^2^ RL = exp((AIC_M−D_ − AIC_M−L_)/2)).

## Data Availability

Restrictions apply to the availability of these data. Data was obtained from Gorzagri (Fonzaso, Italy) and are available from the authors with the permission of Gorzagri.

## Appendix A

**Table A1:** Number of observations and descriptive statistics for the investigated traits.

Trait	Observations	Mean	SD	Min	Max
Average daily gain, kg/d	13,067	0.71	0.07	0.43	1.10
Body weight at 270 d, kg	13,395	166.9	14.8	105.6	226.9
Killing out percentage, %	12,536	82.3	1.4	76.3	85.0
Backfat depth, mm	11,837	26.7	5.2	13.0	49.0
Loin depth, mm	11,307	64.7	6.6	49.0	86.0
Carcass lean meat content, %	11,307	53.4	2.4	42.3	59.7
Average weight of the thigh, kg	12,645	13.9	1.1	10.0	18.0
Raw ham percentage, %	12,620	19.8	0.9	16.1	24.8
Raw ham traits					
Round shape score	13,084	1.8	0.9	0	4
Marbling score	13,083	1.5	0.8	0	4
Subcutaneous fat score	12,945	0.0	1.7	−4	4
Color score	12,946	0.1	1.5	−4	4
Veining score	13,083	1.2	0.9	0	4
Subcutaneous fat depth, mm					
nearby *semimembranosus* muscle	12,909	19.5	5.7	9.0	45.0
nearby *quadriceps femoris* muscle	9314	6.1	1.1	3.0	10.3
Raw ham subcutaneous fat traits					
Fatty acid composition					
SFA, %	1454	35.70	2.87	28.10	44.29
MUFA, %	1454	47.91	1.90	41.36	54.09
PUFA, %	1454	14.87	2.24	8.38	21.64
Unsaturated FA/SFA	1454	1.78	0.22	1.25	2.50
(SFA + MUFA)/PUFA	1452	5.78	1.09	3.43	10.42
C14:0, %	1455	1.39	0.26	0.80	2.05
C16:0, %	1454	22.65	1.69	17.15	26.96
C18:0, %	1454	11.01	1.26	6.97	15.00
C16:1, %	1452	2.39	0.38	1.36	3.88
C18:1n9ct, %	1454	41.07	1.81	35.42	45.67
C18:1n11trans, %	1448	3.22	0.28	2.09	4.47
C18:2n6, %	1454	12.87	1.92	7.24	18.77
ω3, %	1451	0.93	0.17	0.50	1.49
ω6, %	1454	13.38	1.85	7.81	19.30
Iodine number	1449	69.94	3.44	59.30	81.49
Infrared predictions					
Iodine number	12,516	70.29	3.27	58.81	79.78
C18:2n6, %	10,168	13.40	1.70	7.54	19.17
C18:0, %	10,169	10.90	1.50	7.05	16.80
ω6, %	10,167	13.75	1.75	7.87	19.59
PUFA, %	10,169	15.47	1.91	8.79	22.39
MUFA/PUFA, %	10,169	3.21	0.44	1.71	4.80
Dry-curing traits					
Initial ham weight, kg	1707	13.74	1.01	10.55	17.28
Final ham weight, kg	1706	9.92	0.88	6.93	13.19
Ham weight loss, %					
At the end of salting	1700	3.6	0.6	1.5	6.0
From salting to the end of resting	1705	12.4	1.1	8.7	15.9
From resting to the end of curing	1702	14.5	1.9	7.5	22.5
At the end of resting	1704	15.6	1.3	11.4	20.5
At the end of dry-curing	1705	27.8	2.4	20.1	38.2
From salting to end of dry-curing	1705	25.1	2.2	17.3	34.7
Ham weight loss, kg					
At the end of salting	1701	0.50	0.08	0.21	0.86
From salting to the end of resting	1705	1.64	0.14	1.11	2.14
From resting to the end of curing	1704	1.67	0.23	0.94	2.75
At the end of resting	1705	2.13	0.19	1.44	2.84
At the end of dry-curing	1705	3.81	0.35	2.70	5.23
From salting to end of dry-curing	1705	3.31	0.31	2.32	4.60

## References

[1] Toro M.A., Varona L. (2010). A note on mate allocation for dominance handling in genomic selection. Gen. Sel. Evol..

[2] Mäki-Tanila A. (2008). An overview on quantitative and genomic tools for utilising dominance genetic variation in improving animal production. Agric. Food Sci..

[3] Vitezica Z.G., Varona L., Legarra A. (2013). On the Additive and Dominant Variance and Covariance of Individuals Within the Genomic Selection Scope. Genetics.

[4] Varona L., Legarra A., Toro M.A., Vitezica Z.G. (2018). Non-additive Effects in Genomic Selection. Front. Gen..

[5] Gallardo J.A., Lhorente J.P., Neira R. (2010). The consequences of including non-additive effects on the genetic evaluation of harvest body weight in Coho salmon (Oncorhynchus kisutch). Gen. Sel. Evol..

[6] Norris D., Varona L., Ngambi J.W., Visser D.P., Mbajiorgu C.A., Voordewind S.F. (2010). Estimation of the additive and dominance variances in SA Duroc pigs. Livest. Sci..

[7] Nagy I., Farkas J., Curik I., Gorjanc G., Gyovai P., Szendrő Z. (2014). Estimation of additive and dominance variance for litter size components in rabbits. Czech J. Anim. Sci..

[8] Dufrasne M., Faux P., Piedboeuf M., Wavreille J., Gengler N. (2014). Estimation of dominance variance for live body weight in a crossbred population of pigs. J. Anim. Sci..

[9] Prosciutto di Parma (Parma Ham) Protected Designation of Origin (Specifications and Dossier Pursuant to Article 4 of Council Regulation EEC no. 2081/92 dated 14 July 1992). https://www.prosciuttodiparma.com/wp-content/uploads/2019/07/Parma_Ham_Specifications_Disciplinare_Consolidato_Nov_13.pdf.

[10] Costa E.V., Diniz D.B., Veroneze R., Resende M.D.V., Azevedo C.F., Guimarães S.E.F., Silva F.F., Lopes P.S. (2015). Estimating additive and dominance variances for complex traits in pigs combining genomic and pedigree information. Gen. Mol. Res..

[11] Bonfatti V., Carnier P. (2020). Prediction of dry-cured ham weight loss and prospects of use in a pig breeding program. Animal.

[12] Bonfatti V., Boschi E., Carnier P. (2021). On-site visible-near infrared predictions of iodine number and fatty acid composition of subcutaneous fat of raw hams as phenotypes for a heavy pig breeding program. Animal.

[13] Rostellato R., Sartori C., Bonfatti V., Chiarot G., Carnier P. (2015). Direct and social genetic effects on body weight at 270 days and carcass and ham quality traits in heavy pigs. J. Anim. Sci..

[14] Gallo L., Dalla Montà G., Carraro L., Cecchinato A., Carnier P., Schiavon S. (2014). Growth performance of heavy pigs fed restrictively diets with decreasing crude protein and indispensable amino acids content. Livest. Sci..

[15] AOAC (1980). Official Methods of Analysis.

[16] Riovanto R. (2011). Near Infrared Spectroscopy in Food Analysis: Qualitative and Quantitative Approaches. Ph.D. Thesis.

[17] Misztal I., Tsuruta S., Strabel T., Auvray B., Druet T., Lee D. BLUPF90 and related programs (BGF90). Proceedings of the 7th World Congress on Genetics Applied to Livestock Production.

[18] Arango J., Misztal I., Tsuruta S., Culbertson M., Herring W. (2005). Estimation of variance components including competitive effects of Large White growing gilts. J. Anim. Sci..

[19] Buttazzoni L., Gallo M., Baiocco C., Carchedi C. (1993). La selezione per la qualità della carne suina destinata alla trasformazione. Riv. Suinic..

[20] Carnier P., Cassandro M., Knol E., Padoan D. Genetic parameters for some carcass traits and fresh ham traits in crossbred Goland pigs. Proceedings of the XIII ASPA Congress.

[21] ANAS (2016). ANAS Notizie: La Selezione Per Il Prosciutto DOP. Una Sfida Tecnica Per Un Orizzonte di Lungo Periodo..

[22] Serenius T., Stalder K.J., Puonti M. (2006). Impact of dominance effects on sow longevity. J. Anim. Breed. Gen..

[23] Misztal I., Besbes B. (2000). Estimates of parental-dominance and full-sib permanent environment variances in laying hens. Anim. Sci..

[24] Guo X., Christensen O.F., Ostersen T., Wang Y., Lund M.S., Su G. (2016). Genomic prediction using models with dominance and imprinting effects for backfat thickness and average daily gain in Danish Duroc pigs. Gen. Sel. Evol..

[25] Lopes M.S., Bastiaansen J.W.M., Janss L., Knol E.F., Bovenhuis H. (2015). Genomic prediction of growth in pigs based on a model including additive and dominance effects. J. Anim. Breed. Gen..

[26] Culbertson M.S., Mabry J.W., Misztal I., Gengler N., Bertrand J.K., Varona L. (1998). Estimation of dominance variance in purebred Yorkshire swine. J. Anim. Sci..

[27] González-Diéguez D., Tusell L., Carillier-Jacquin C., Bouquet A., Vitezica Z.G. (2019). SNP-based mate allocation strategies to maximize total genetic value in pigs. Gen. Sel. Evol..

[28] Tusell L., Gilbert H., Vitezica Z.G., Mercat M.J., Legarra A., Larzul C. (2019). Dissecting total genetic variance into additive and dominance components of purebred and crossbred pig traits. Animal.

[29] Varona L., Misztal I., Bertrand J.K., Lawlor T.J. (1998). Effect of full-sibs on additive breeding values under the dominance model for stature in United States Holstein. J. Dairy Sci..

[30] Huang W., Mackay T.F.C. (2016). The Genetic Architecture of Quantitative Traits Cannot Be Inferred from Variance Component Analysis. PLoS Genet..

[31] Mrode R.A. (2014). Linear Models for the Prediction of Animal Breeding Values.

[32] Lee S.H., Goddard M.E., Visscher P.M., van der Werf J.H. (2010). Using the realized relationship matrix to disentangle confounding factors for the estimation of genetic variance components of complex traits. Gen. Sel. Evol..

